# Fabrication of Nitrogen-Doped Reduced Graphene Oxide Modified Screen Printed Carbon Electrode (N-rGO/SPCE) as Hydrogen Peroxide Sensor

**DOI:** 10.3390/nano12142443

**Published:** 2022-07-16

**Authors:** Khursheed Ahmad, Haekyoung Kim

**Affiliations:** School of Materials Science and Engineering, Yeungnam University, Gyeongsan 38541, Korea; khursheed.energy@gmail.com

**Keywords:** nitrogen-doped reduced graphene oxide, screen printed carbon electrode, N-rGO/SPCE, hydrogen peroxide sensor

## Abstract

In recent years, the electrochemical sensing approach has attracted electrochemists because of its excellent detection process, simplicity, high sensitivity, cost-effectiveness, and high selectivity. In this study, we prepared nitrogen doped reduced graphene oxide (N-rGO) and characterized it using various advanced techniques such as XRD, SEM, EDX, Raman, and XPS. Furthermore, we modified the active surface of a screen printed carbon electrode (SPCE) via the drop-casting of N-rGO. This modified electrode (N-rGO/SPCE) exhibited an excellent detection limit (LOD) of 0.83 µM with a decent sensitivity of 4.34 µAµM^−1^cm^−2^ for the detection of hydrogen peroxide (H_2_O_2_). In addition, N-rGO/SPCE also showed excellent selectivity, repeatability, and stability for the sensing of H_2_O_2_. Real sample investigations were also carried out that showed decent recovery.

## 1. Introduction

Hydrogen peroxide (H_2_O_2_) is a well-known by-product of various oxidase enzymes and is an oxidizing agent [1,2]. H_2_O_2_ plays a crucial role in the production of organic compounds, textiles, and is also used as bleaching agent in the food industries [3]. H_2_O_2_ may also control many biological processes such as immune cell activation, vascular remodeling, and root growth in living organisms [4]. Despite the extensive use of H_2_O_2_ in various fields, a high concentration of H_2_O_2_ may cause some diseases such as diabetes and Parkinson’s disease [5]. H_2_O_2_ may also cause skin or eye irritation, cancer, itching, and cardiovascular disease [6]. Thus, there is an immense interest and demand in developing a cost-effective, selective, highly sensitive, and stable H_2_O_2_ sensor [7,8,9,10]. In this connection, various conventional methods such as fluorescence, spectrophotometry, chromatography, and colorimetric methods have been used for the detection of H_2_O_2_ [11,12,13,14,15]. In the past few years, electrochemical methods have received huge interest because of their low cost, simple fabrication, high sensitivity, selectivity, and stability [16,17,18,19,20,21]. Previously, electrochemical methods have been widely used for the detection of hazardous/toxic compounds or biomolecules [22,23,24,25,26].

In this connection, nickel oxide (NiO)/carbon foam was applied as a H_2_O_2_ sensor by Liu et al. [20], which exhibited a limit of detection (LOD) of 0.013 µM. Ahmad et al. [2] reported MgO/SPE as a H_2_O_2_ sensor that demonstrated a LOD of 0.3 µM. Karuppiah et al. [27] utilized novel strategies and reported a LOD of 0.27 µM while Dang et al. [24] obtained a LOD of 1.2 µM. In other work, Uzunoglu et al. [28] developed a cerium dioxide (CeO_2_) modified electrode as a H_2_O_2_ sensor and reported a LOD of 2 µM. Graphene was also reported as a electrode modifier for the construction of a H_2_O_2_ sensor [29]. The fabricated sensor showed a LOD of 6.5 µM [29]. In other reports, an excellent LOD of 0.08 µM was obtained by Jiang et al. [30] and Chen et al. [31]. A new electrode material of zinc manganese oxide (ZnMn_2_O_4_) was also utilized as an electrode material by Li et al. [32], which showed a decent LOD of 0.13 µM. A hybrid composite of nickel cobalt sulfide and graphene (NiCo_2_S_4_/graphene) was also employed as an electrode modifier for the fabrication of a H_2_O_2_ sensor [33]. Thus, it can be understood that the electrochemical sensing ability of the developed H_2_O_2_ sensor can be affected by the physiochemical properties of the electrode materials. The previous literature also showed that electrode materials should have an excellent conductive nature as well as electro-catalytic properties [34,35,36,37].

Reduced graphene oxide (rGO) is a material of interest because of its excellent attributes such as high chemical stability, mechanical property, high surface area, electrical conductivity, and unique electronic properties [36]. rGO has a two-dimensional (2D) honeycomb lattice with single layer of carbon atoms [37,38,39]. Previously, rGO has been widely explored in various applications such as dye sensitized solar cells, electrochemical sensors, catalysis, batteries, supercapacitors, and perovskite solar cells. rGO has also been used to improve the electrical conductivity of poor semiconducting metal oxides [40,41]. In the last few years, the functionalization or modification of rGO has received extensive attention [42]. Previous reports have shown that the introduction of heteroatoms into the rGO matrix can cause defects, tune surface active sites, and tailor its band structure [43].

Herein, we prepared a nitrogen (N) doped rGO (N-rGO) using the hydrothermal method and a screen printed carbon electrode (SPCE) was modified with N-rGO as the electrode material for H_2_O_2_ sensing.

## 2. Materials and Methods

### 2.1. Chemicals and Reagents

Chemicals/reagents, solvents, and phosphate buffer solutions were purchased from Alfa Aesar, Merck, TCI, Fisher Scientific, and Sigma, Missouri, MO, USA.

### 2.2. Synthesis of N@rGO

Graphene oxide (GO) was prepared using a previous report [44]. To prepare the N-rGO, GO (50 mg) was added to 50 mL of distilled water (DW) and sonicated for 2 h. Furthermore, an appropriate amount of urea was added to the GO solution. Finally, this prepared aqueous solution of GO was poured into the Teflon-lined stainless steel autoclave cup (capacity 100 mL) and heated at 200 °C for 6 h in a vacuum furnace. The obtained precipitate was collected by centrifugation and washed with DW and ethanol several times to remove the residual impurities. Finally, the obtained powder was dried at 60 °C overnight and labeled as N-rGO.

### 2.3. Fabrication of N-rGO/SPCE

N-rGO (3 mg) was dispersed in 1.5 mL of DW and sonicated for 1 h. Furthermore, 7.5 µL of N-rGO was deposited on the active surface of SPCE to construct the H_2_O_2_ sensor. 

**Scheme 1:**
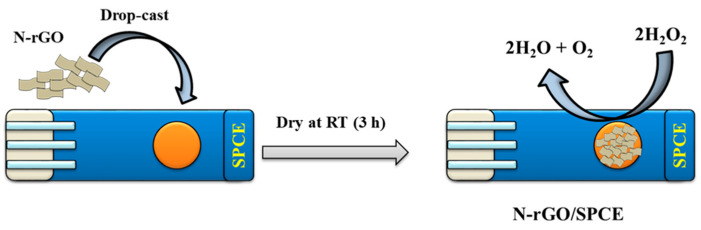
The schematic illustration for the fabrication of N-rGO/SPCE for the H_2_O_2_ sensor.

The N-rGO modified SPCE (N-rGO/SPCE) was dried in air for 3 h at room temperature (RT). The modification of SPCE with N-rGO is illustrated in Scheme 1. The electrochemical sensing properties of N-rGO/SPCE were examined on a computer controlled three-electrode CH instrument using N-rGO/SPCE as the working electrode whereas a platinum electrode acted as the counter electrode. A silver/silver chloride (Ag/AgCl) electrode was used as the reference electrode.

## 3. Results

### 3.1. Physiochemical Characterization of N-rGO

**Figure 1:**
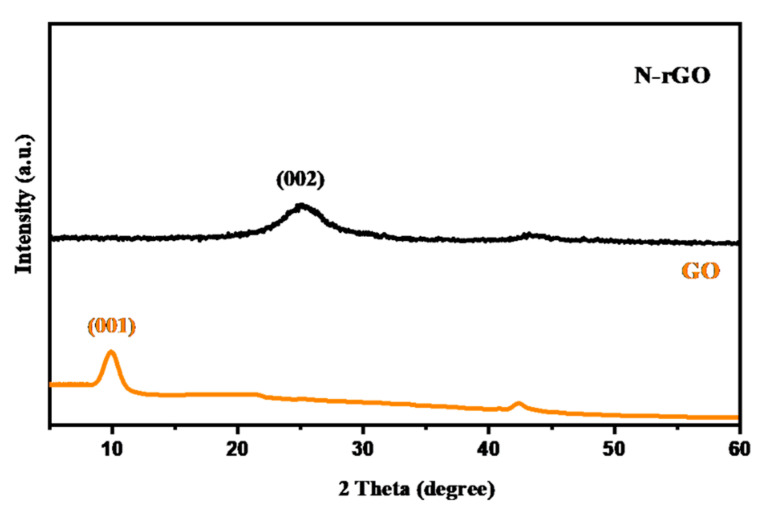
The XRD pattern of GO and N-rGO.

The powder X-ray diffraction (XRD) patterns of the GO and N-rGO were obtained on an X’pert Pro X-ray diffractometer. The recorded XRD pattern of the GO and N-rGO is displayed in Figure 1. The XRD pattern of GO showed a strong diffraction peak at ~10.1°, which can be assigned to the (001) diffraction plane of GO. In the case of N-rGO, a broad diffraction peak appeared at ~25.34°. This diffraction peak was attributed to the (002) diffraction plane and suggested the successful transformation of GO to N-rGO in the presence of urea (as N source). No other diffraction peak appeared in the XRD pattern of N-rGO, which suggests its phase purity. The obtained results are in well-agreement with previous studies [43].

Scanning electron microscopy (SEM, Hitachi, S-4800) was further adopted to characterize the surface morphological properties of the GO and N-rGO.

**Figure 2:**
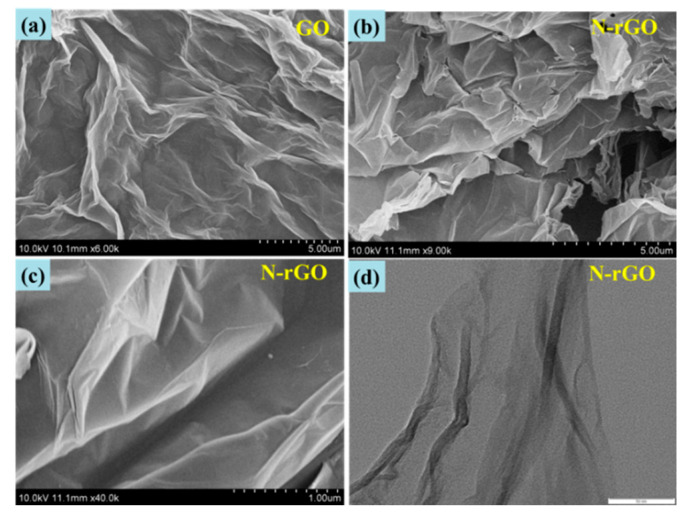
The SEM image of the GO (**a**) and N-rGO (**b**,**c**). The TEM image of N-rGO (**d**).

**Figure 3:**
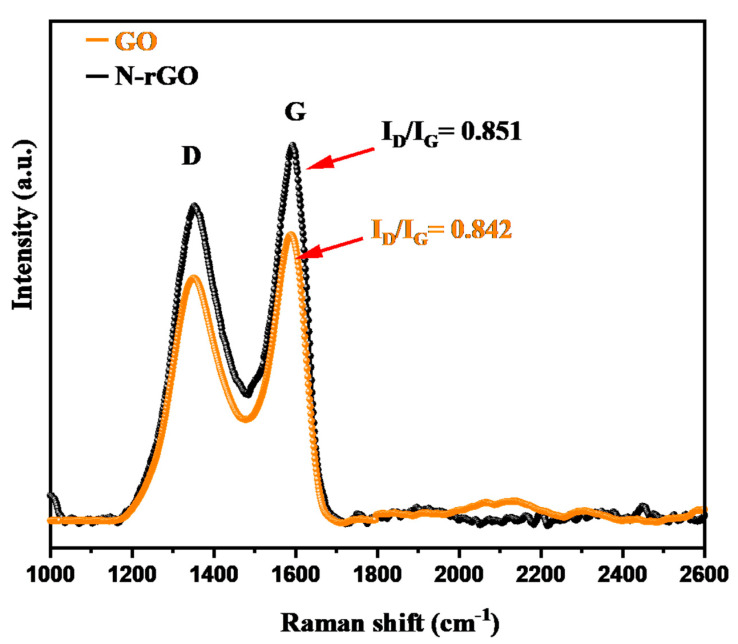
The Raman spectra of GO and N-rGO.

Figure 2a showed that prepared GO has a sheet-like surface morphology. In the case of N-rGO, a wrinkled sheet-like surface was observed (Figure 2b,c). The presence of the wrinkled sheet-like surface morphology of N-rGO may be due to the hydrothermal treatment. The obtained morphological characteristics of GO and N-rGO were well-matched with previous studies [42]. Figure 2d displays the transmission electron microscopic (TEM) image of N-rGO, which suggests that N-rGO consists of wrinkled sheet-like surface. The Raman spectroscopic investigation were performed on a Horiba scientific instrument and the recorded Raman data of the prepared GO and N-rGO are displayed in Figure 3. The Raman data of GO and N-rGO exhibited two-vibrational modes that can be assigned as D and G bands in the GO and N-rGO samples. The Raman study was consistent with previous studies [45,46]. The I_D_/I_G_ ratio of the GO and N-rGO is presented in Figure 3. The observations suggest that the prepared N-rGO has a graphitic nature with low-defects.

**Figure 4:**
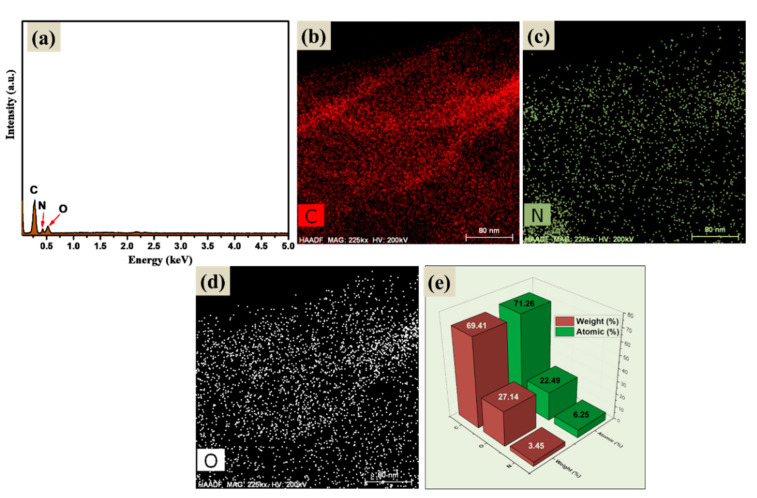
The EDX spectrum (**a**), EDX mapping images (**b**–**d**), and elemental composition (**e**) of N-rGO.

**Figure 5:**
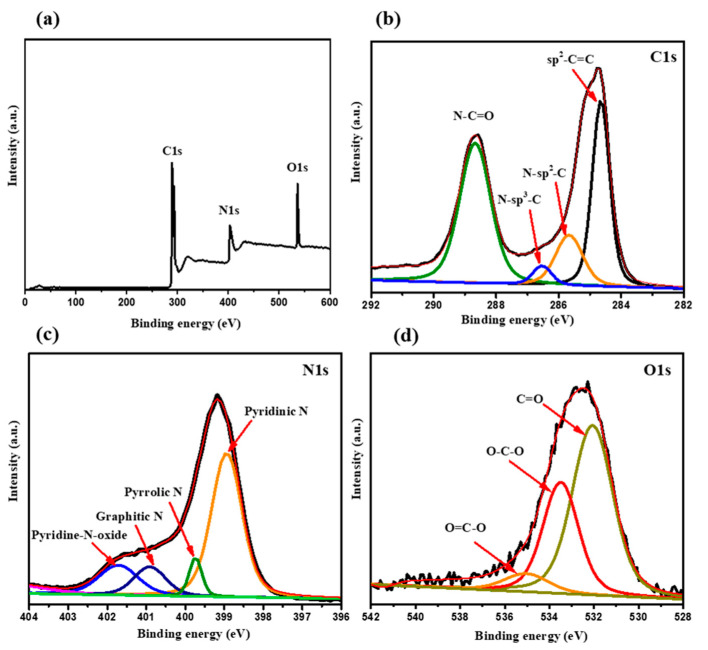
The XPS survey scan (**a**), C1s (**b**), N1s (**c**), and O1s (**d**) scan of N-rGO.

The presence of N in the N-rGO was confirmed by using energy-dispersive X-ray (EDX) spectroscopy. The EDX spectrum and EDX mapping images of the N, C, and O elements are displayed in Figure 4a–d. The elemental composition of N-rGO is provided in Figure 4e. The EDX spectrum confirmed the presence of N in the prepared N-rGO (Figure 4a). Subsequently, we used X-ray photoelectron spectroscopy (XPS, Thermo Scientific Instrument) to investigate the binding energy and oxidation states of the N, C and O elements in the N-rGO sample. Figure 5a depicts the XPS survey scan of N-rGO, which clearly shows the presence of the N, C, and O elements. The C1 scan of N-rGO is presented in Figure 5b, which shows four peaks. These peaks can be assigned to N-C=O, N-sp^3^-C, N-sp^2^-C, and sp^2^-C=C [47]. Figure 5c shows the N1s scan of N-rGO, which shows the presence of pyridinic N, pyrrolic N, graphitic N, and pyridine-N-oxide [47]. The reported literature suggests that pyridinic N can be more favorable for the enhancement of the electrochemical properties of N-rGO/GCE than pyrrolic N [47]. The obtained results confirmed the presence of N in the N-rGO sample and is consistent with previous study [47,48]. Figure 5d depicts the O1s scan of N-rGO, which indicates the presence of O=C-O, O-C-O, and C=O bonds [47]. The atomic weight percentage of C1s, N1s, and O1s elements were found to be 65.21%, 3.66%, and 31.13%, respectively, using the XPS method.

### 3.2. Electrochemical Performance of N-rGO/SPCE

**Figure 6:**
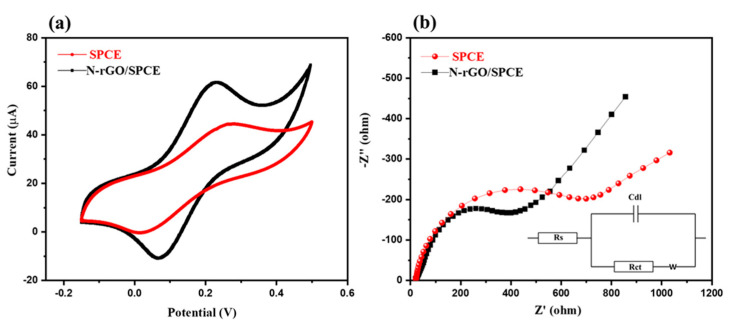
CV (**a**) and Nyquist (**b**) curves of SPCE (red) and N-rGO/SPCE (black) in the 3.0 mM K_3_ [Fe(CN)_6_] and K_4_[Fe(CN)_6_] redox solution in 0.1 M KCl. Inset of (**b**) shows the fitted equivalent circuit.

The electro-catalytic properties of the N-rGO/SPCE and bare SPCE were investigated in the K_3_[Fe(CN)_6_] and K_4_[Fe(CN)_6_] redox system. Figure 6a shows the cyclic voltammograms (CV) of the N-rGO/SPCE and bare SPCE in the presence of 3 mM K_3_[Fe(CN)_6_]/K_4_[Fe(CN)_6_] redox solution in 0.1 M PBS (pH = 7.0) at a scan rate of 50 mVs^−1^. Observations indicated that N-rGO/SPCE has a higher current response compared to the bare SPCE (Figure 6a). This higher electro-catalytic activity of N-rGO/SPCE may be attributed to the better electro-catalytic properties of the N-rGO. Furthermore, electrochemical impedance spectroscopy (EIS) was also used to examine the charge kinetics of the N-rGO/SPCE and bare SPCE. Figure 6b shows the Nyquist plot of the N-rGO/SPCE and bare SPCE in the 3 mM K_3_[Fe(CN)_6_]/K_4_[Fe(CN)_6_] redox solution in 0.1 M PBS (pH = 7.0). The fitted equivalent circuit is inserted in the inset of Figure 6b. The obtained data showed that the bare SPCE had a larger semi-circle with a high charge-transfer resistance (Rct) of 749.4 Ω. The N-rGO/SPCE showed a lower Rct value of 487.2 Ω with a relatively small semicircle (Figure 6b).

In addition, the EIS curves of the SPCE and N-rGO/SPCE showed a difference in the low-frequency region, which can be related to the diffusion process. From the obtained EIS data, it can be said that N-rGO/SPCE had a better electro-catalytic ability compared to the bare SPCE.

**Figure 7:**
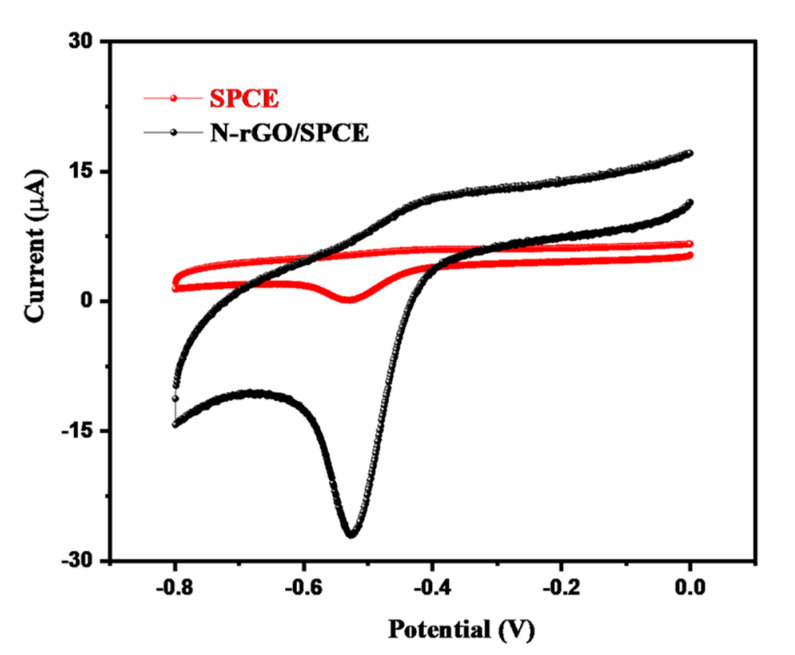
The CV curves of SPCE (red) and N-rGO/SPCE (black) in the presence of 0.01 mM H_2_O_2_ in 0.1 M PBS of pH 7.0 at a scan rate of 50 mVs^−1^.

Furthermore, we investigated the electrochemical properties of the N-rGO/SPCE bare SPCE toward the detection of H_2_O_2_. Figure 7 shows the CV curves of the N-rGO/SPCE and bare SPCE in the presence of 0.01 mM H_2_O_2_ in 0.1 M PBS (pH 7.0, scan rate = 50 mVs^−1^). A poor current response was observed for the bare SPCE whereas an interesting and enhanced current response was obtained for N-rGO/SPCE toward the reduction of H_2_O_2_. This enhanced current response for the reduction of H_2_O_2_ was attributed to the excellent electro-catalytic features of N-rGO.

**Figure 8:**
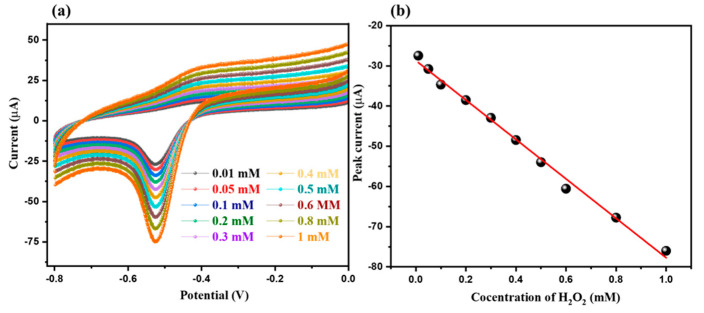
The CV curves (**a**) of N-rGO/SPCE in the presence of different concentrations of H_2_O_2_ (0.01, 0.05, 0.1, 0.2, 0.3, 0.4, 0.5, 0.6, 0.8, and 1.0 mM) in 0.1 M PBS of pH 7.0 at a scan rate of 50 mVs^−1^ and the corresponding linear calibration plot (**b**) between the peak current response versus the concentration of H_2_O_2_.

The effect of leading amount of N-rGO was also optimized and the obtained results are presented in Figure S1. The observations revealed that the highest current response was obtained for 7.5 µL N-rGO loaded SPCE (Figure S1). The pH of the solution may also influence the electrochemical performance of the N-RGO/SPCE. Hence, we recorded the CV graphs of the N-rGO/SPCE in the presence of 0.01 mM H_2_O_2_ in 0.1 M PBS with different pH (3.0, 5.0, 7.0, 9.0, and 11.0) at a scan rate of 50 mVs^−1^. The obtained results are depicted in Figure S2. Figure S2 revealed that N-rGO/SPCE had higher electro-catalytic properties in a PBS of pH 7.0. Thus, we used PBS of pH 7.0 for further electrochemical investigations. The influence of the various concentrations of H_2_O_2_ on the electrochemical performance of N-rGO/SPCE was also studied using CV. We obtained different CV curves of N-rGO/SPCE in the presence of different concentrations of H_2_O_2_ of 0.01 mM to 1 mM at the applied scan rate of 50 mVs^−1^. The recorded CV curves of N-rGO/SPCE in different concentrations of H_2_O_2_ are summarized in Figure 8a. The observations clearly showed that the reduction peak current responses increased with an increasing concentration of H_2_O_2_. This enhanced current response was found to be linear, as suggested by the calibration plot between the current response versus concentration of H_2_O_2_ (Figure 8b). The CV curves of the bare SPCE were also recorded in the presence of various concentrations (0.01 mM to 1 mM) of H_2_O_2_ at a scan rate of 50 mVs^−1^. The current response increased with the increasing concentration of H_2_O_2_ (Figure S3a), but the obtained current response was lower than that of N-rGO/SPCE. The calibration plot is presented in Figure S3b.

**Figure 9:**
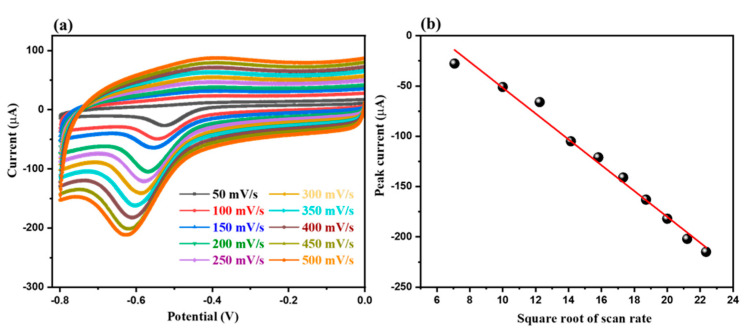
The CV curves (**a**) of N-rGO/SPCE in the presence 0.01 mM H_2_O_2_ in 0.1 M PBS of pH 7.0 at different applied scan rates (50–500 mVs^−1^) and the corresponding linear calibration plot (**b**) between the peak current response versus the square root of the scan rate.

Furthermore, we also investigated the impact of different scan rates on the electro-catalytic properties of the N-rGO/SPCE toward the reduction of H_2_O_2_. Figure 9a shows the obtained CV curves of the N-rGO/SPCE in the presence of 0.01 mM at different applied scan rates of 50–500 mVs^−1^. We observed that the reduction peak current response for the reduction of H_2_O_2_ increased with an increasing scan rate of 50–500 mVs^−1^. We calibrated the reduction peak current responses with respect to the square root of the scan rate, and the calibration curve is depicted in Figure 9b. This calibration curve suggests that the reduction in peak current response linearly increases (Figure 9b). We also recorded the CV graphs of the bare SPCE in the presence of 0.01 mM H_2_O_2_ in 0.1 M PBS of pH 7.0 at different applied scan rates (50–500 mVs^−1^). The obtained CV results are presented in Figure S4a. The observations revealed that the current response increased with the increasing scan rate but the obtained current response was found to be less when compared to that of the N-rGO/SPCE. Moreover, the obtained current response was found to be nonlinear (Figure S4b).

**Figure 10:**
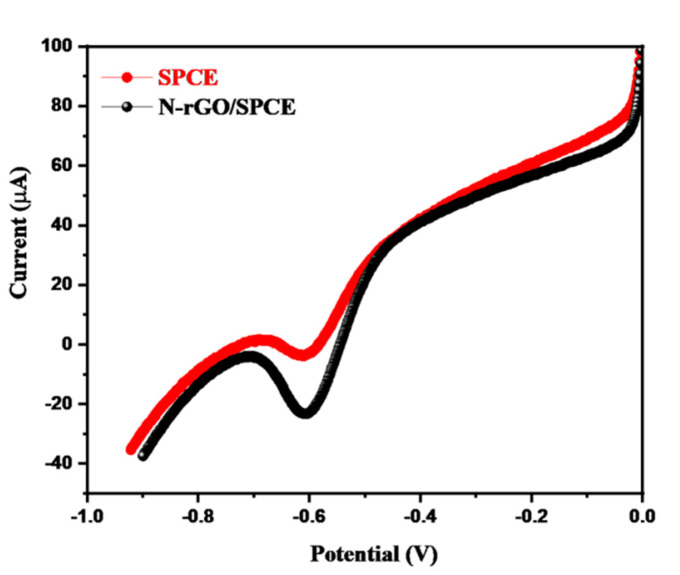
The LSV curves of SPCE (red) and N-rGO/SPCE (black) in the presence of 0.01 mM H_2_O_2_ in 0.1 M PBS of pH 7.0 at a scan rate of 50 mVs^−1^.

In the past few years, the linear sweep voltammetry (LSV) method has been extensively used for the determination of various toxic, hazardous, or biomolecules. Thus, we also further adopted the LSV technique to detect the H_2_O_2_. Figure 10 shows the LSV curves of the N-rGO/SPCE and bare SPCE in the presence of 0.01 mM H_2_O_2_ in 0.1 M PBS (pH 7.0, scan rate = 50 mVs^−1^). The LSV results showed that the bare SPCE has poor electro-catalytic activity compared to the N-rGO/SPCE for the reduction of H_2_O_2_ (Figure 10). This improvement in the reduction peak current response for the reduction of H_2_O_2_ was attributed to the presence of the excellent electro-catalytic properties in N-rGO.

**Figure 11:**
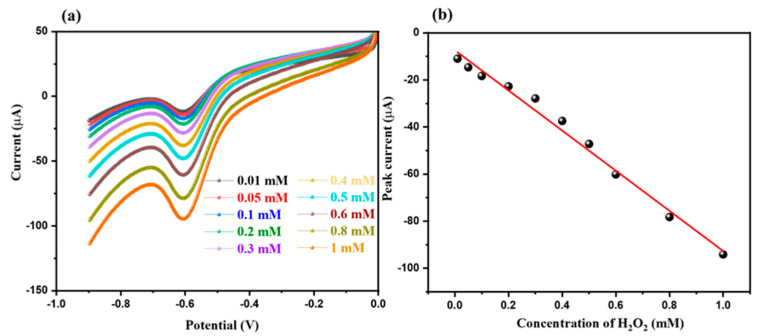
The LSV curves (**a**) of N-rGO/SPCE in the presence of different concentrations of H_2_O_2_ (0.01, 0.05, 0.1, 0.2, 0.3, 0.4, 0.5, 0.6, 0.8, and 1.0 mM) in 0.1 M PBS of pH 7.0 at a scan rate of 50 mVs^−1^ and the corresponding linear calibration plot (**b**) between the peak current response versus the concentration of H_2_O_2_.

Similar to the CV results, we also studied the influence of various concentrations of H_2_O_2_ on the electrochemical performance of the fabricated N-rGO/SPCE using the LSV method. The recorded LSV curves of the N-rGO/SPCE in the presence of various concentrations of H_2_O_2_ (0.01 mM to 1 mM) at the applied scan rate of 50 mVs^−1^ are displayed in Figure 11a. This study showed that the reduction peak current response increased with an increasing concentration of H_2_O_2_. The calibration plot between the reduction peak current responses versus concentrations of H_2_O_2_ is plotted in Figure 11b, which shows that the current response linearly increased. Figure 11b shows the acceptable linear-regression value of −83.583x − 7.7861 and the correlation-coefficient (R^2^) value of 0.979.

**Figure 12:**
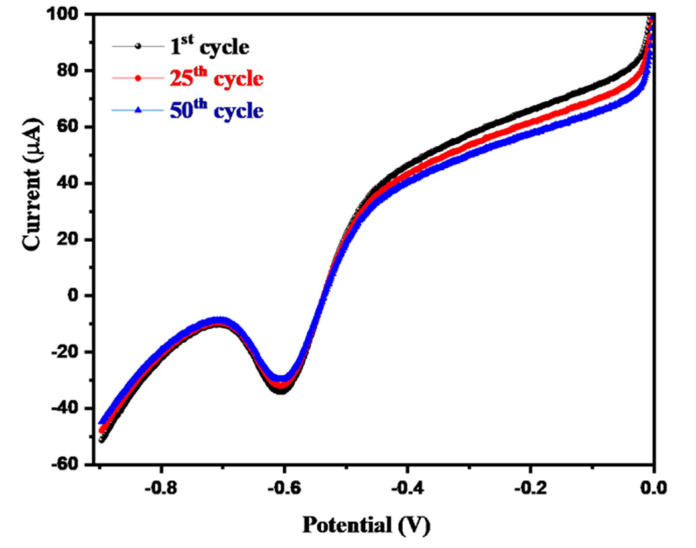
The LSV curves (1^st^, 25^th^, and 50^th^ cycle) of N-rGO/SPCE in the presence of 0.05 mM H_2_O_2_ in 0.1 M PBS of pH 7.0 at a scan rate of mVs^−1^.

Furthermore, we also recorded 50 consecutive LSV curves of N-rGO/SPCE in the presence of 0.05 mM H_2_O_2_ at the applied scan rate of 50 mVs^−1^. The 1^st^, 25^th^, and 50^th^ LSV curves of the N-rGO/SPCE are presented in Figure 12.

**Figure 13:**
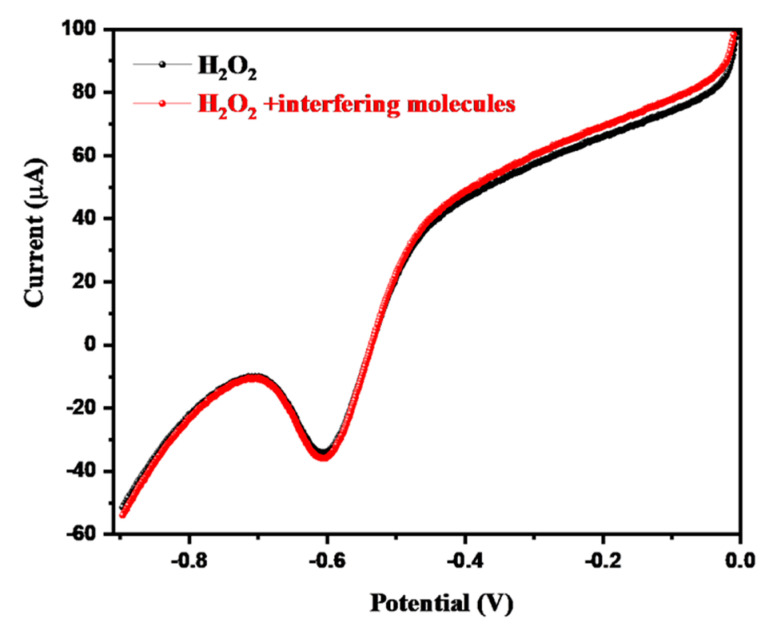
The LSV curves of N-rGO/SPCE in the absence (black) and presence (red) of interfering molecules (glucose, chlorophenol, hydrazine, uric acid, urea, dopamine, ascorbic acid, and nitrophenol) in 0.05 mM H_2_O_2_ in 0.1 M PBS of pH 7.0 at a scan rate of 50 mVs^−1^.

The obtained LSV curves showed an insignificant variation in the reduction peak current response up to the 50^th^ cycle. This suggested excellent cyclic stability and repeatability up to 50 cycles. The selectivity of the electrochemical sensors is the most important feature for practical purposes. In this connection, we investigated the selectivity of the fabricated N-rGO/SPCE in the absence and presence of various interfering molecules (glucose, chlorophenol, hydrazine, uric acid, urea, dopamine, ascorbic acid, and nitrophenol) in 0.05 mM H_2_O_2_ (conditions: 0.1 M PBS, pH = 7.0, scan rate = 50 mVs^−1^) using the LSV method. Figure 13 shows the LSV curves of N-rGO/SPCE in the absence (black) and presence (red) of various interfering molecules (glucose, chlorophenol, hydrazine, uric acid, urea, dopamine, ascorbic acid, and nitrophenol) in 0.05 mM H_2_O_2_. An insignificant change was observed in the presence of various interfering molecules, which suggested the excellent selective nature of the N-rGO/SPCE toward the sensing of H_2_O_2_. We also checked the selectivity test using bare SPCE under similar conditions and the obtained results are presented in Figure S5. The observations showed that bare SPCE is not suitable for the selective detection of H_2_O_2_ (Figure S5). During the detection process, H_2_O_2_ reduces and releases H_2_O and O_2_. The probable mechanism for the reduction of H_2_O_2_ is illustrated in Scheme 1 [22].

Furthermore, we also employed chronoamperometry to detect H_2_O_2_ using N-rGO/SPCE. The obtained results are presented in Figure S6a. The obtained results showed that the amperometric signal (in terms of current response) increased with each spike of H_2_O_2_ (Figure S6a). This increased current response was found to be linear, as confirmed by the calibration plot between the current response and the concentration of H_2_O_2_ (Figure S6b). Figure S6b shows the acceptable linear-regression value of −0.0894x + 0.18 and correlation-coefficient (R^2^) value of 0.998.

The selectivity test was also carried out using chronoamperometry. Figure S7 shows that the current response increased with the addition of H_2_O_2_, but the addition of interfering molecules did not affect the performance of the N-rGO/SPCE. Therefore, it can be understood that N-rGO/SPCE has good selectivity toward the detection of H_2_O_2_.

The electrochemical performance of the fabricated sensors can be evaluated in terms of the sensitivity and limit of detection (LOD). In this regard, we calculated the LOD and sensitivity of the N-rGO/SPCE for the sensing of H_2_O_2_ using Equations (1) and (2), which are given below [22]:

LOD = 3.3 (σ/S)(1)

Sensitivity = S/area of the working electrode(2)

(In Equation (1), σ = standard-error whereas and S = slope).

The calculated sensitivity and LOD of the N-rGO/SPCE toward the sensing of H_2_O_2_ using the CV and LSV methods are presented in Table 1. The obtained results are comparable with previous reports as listed in Table 1 [49,50,51,52,53,54,55,56,57,58,59,60,61,62].

In previous years, various electrode materials have been explored for the construction of H_2_O_2_ sensors. In this connection, Zhao et al. [49] fabricated a graphene/silver (Ag) electrode and reported a LOD of 2.8 µM. Du et al. [50] reported platinum (Pt)/polyaniline (PANI)/rGO as a H_2_O_2_ sensor that exhibited a LOD of 1.1 µM. In 2021, Promsuwan et al. [51] developed a H_2_O_2_ sensor using a palladium (Pd)/PANI/carbon microsphere (CMF) electrode that showed an excellent LOD of 0.70 µM. Yin et al. [52] used a copper gallate spinel (CuGa_2_O_4_) modified GCE as a H_2_O_2_ sensor and obtained a LOD of 5 µM. Bohlooli et al. [53] developed a manganese oxide (MnOx)/carbon nanowall based non-enzymatic H_2_O_2_ sensor whereas Xiao et al. [54] developed a H_2_O_2_ sensor and reported on MnO_2_-carbon nanofibers (CNF)/GCE as the H_2_O_2_ sensor. In other work, copper bismuth oxide was prepared on a fluorine doped tin oxide (FTO) substrate and used as a H_2_O_2_ sensor, which exhibited a LOD of 380 µM [55]. A hybrid composite of molybdenum disulfide (MoS_2_) and rGO was also explored for H_2_O_2_ sensing applications by Yang et al. [56]. AuNP-Prussian blue (PB)-graphene oxide (GO) was used as a H_2_O_2_ sensor by Liu et al. [57] while CuNP-rGO was applied as a H_2_O_2_ sensor by Nia et al. [58]. Mutyala et al. [58] prepared electrochemically reduced graphene oxide (ERGO) using the electrodeposition approach and fabricated a H_2_O_2_ sensor that showed a LOD of 0.7 µM. Woo et al. [60] and Liu et al. [61] also reported a H_2_O_2_ sensor using graphene/MWCNT and Ag/rGO as the electrode modifier, respectively. Karimi et al. [62] reported the facile synthesis of a rGO/iron oxide (Fe_2_O_3_) composite for the construction of H_2_O_2_ sensor. Our obtained results are comparable with previous reports in terms of the LOD (Table 1).

## 4. Conclusions

Finally, it can be summarized that N-rGO was prepared using the hydrothermal method. The formation of N-rGO was authenticated by the XRD method whereas the phase purity of the N-rGO was checked by the XPS technique. The presence of N in the N-rGO sample was confirmed by the EDX and XPS techniques. Furthermore, a screen printed carbon electrode (SPCE) was fabricated using N-rGO as the electrode modifier. This fabricated N-rGO/SPCE was applied as the working electrode for the sensing of H_2_O_2_. The obtained electrochemical results for N-rGO/SPCE exhibited an excellent detection limit and decent sensitivity. N-rGO/SPCE also showed good cyclic stability, repeatability, and selectivity for the detection of H_2_O_2_. We hope that N-rGO/SPCE can be explored for practical applications because of its low cost, stability, selectivity, and high performance.

## Figures and Tables

**Table 1:** The comparisons of the LOD and sensitivity with previous reports.

Electrode Material	LOD (µM)	Sensitivity	Working Electrode Area	References
Graphene@Ag	2.8	28.6 µM mM^−1^ cm^−2^	5 mm	[49]
Pt-PANI/rGO	1.1	257 μA mM^−1^ cm^−2^	-	[50]
Pd/PANI/(CMF)	0.70	234 μA mM^−1^ cm^−2^	-	[51]
CuGa_2_O_4_/GCE	5	-	3 mm	[52]
MnOx/carbon nanowall	0.55	698 μA mM^−1^ cm^−2^	10 mm	[53]
MnO_2_-CNF/GCE	1.1	71 μA mM^−1^ cm^−2^	3 mm	[54]
CuBi_2_O_4_/FTO	380	280 μA mM^−1^ cm^−2^	0.5 cm^2^	[55]
MoS_2_/rGO	0.19		3 mm	[56]
AuNPs-PB-GO	1.3	7.6 μA mM^− 1^ cm^−2^	-	[57]
CuNP-rGO	600	-	-	[58]
ERGO/GC	0.7	-	-	[59]
Graphene/MWCNT	9.4	-	3 mm	[60]
Ag/rGO	28	-	-	[61]
rGO/Fe_2_O_3_	6	-	2 mm	[62]
N-rGO/SPCE	0.83	4.34 µA µM^−1^ cm^−2^	3 mm	**This study**

## Data Availability

Not applicable.

## Supplementary Materials

The following supporting information can be downloaded at: https://www.mdpi.com/article/10.3390/nano12142443/s1, Figure S1: Effect of loading amount of N-rGO on SPCE surface in presence of 0.01 mM H_2_O_2_ in 0.1 M PBS of pH 7.0 at scan rate of 50 mVs^−1^. Figure S2: CV curves of the N-rGO/SPCE in presence of 0.01 mM H_2_O_2_ in 0.1 M PBS of different pH (3,5,7,9, and 11) at scan rate of 50 mVs^−1^. Figure S3: CV curves (a) of SPCE in presence of different concentration of H2O2 (0.01, 0.05, 0.1, 0.2, 0.3, 0.4, 0.5, 0.6, 0.8, and 1.0 mM) in 0.1 M PBS of pH 7.0 at scan rate of 50 mVs^−1^ and corresponding linear calibration plot (b) between peak current response versus concentration of H_2_O_2_. Figure S4: CV curves (a) of SPCE in presence 0.01 mM H_2_O_2_ in 0.1 M PBS of pH 7.0 at different applied scan rates (50-500 mVs^−1^) and corresponding linear calibration plot (b) between peak current response versus square root of scan rate. Figure S5: LSV curves of SPCE in absence (black) and presence (red) of interfering molecules (glucose, chlorophenol, hydrazine, uric acid, urea, dopamine, ascorbic acid, and nitro-phenol) in 0.05 mM H_2_O_2_ in 0.1 M PBS of pH 7.0 at scan rate of 50 mVs^−1^. Figure S6: Chronoamperometry curve (a) of N-rGO/SPCE in presence of different concentration of H_2_O_2_. Calibration curve (b) between current response versus concentration of H_2_O_2_. Figure S7: Selectivity test of N-rGO/SPCE towards H_2_O_2_ determination in presence of various interfering molecules.
