# Supplementary material for: Fabrication of Nitrogen-Doped Reduced Graphene Oxide Modified Screen Printed Carbon Electrode (N-rGO/SPCE) as Hydrogen Peroxide Sensor
Source: Nanomaterials (Basel). 2022 Jul 16;12(14):2443. doi: 10.3390/nano12142443 (PMC9324769; doi:10.3390/nano12142443)
Supplement: Supplementary file 1 [file nanomaterials-12-02443-s001.zip › nanomaterials-1785223-supplementary.pdf]

Supplementary information

Fabrication of Nitrogen-Doped Reduced Graphene Oxide Modified Screen Printed Carbon Electrode (N-rGO/SPCE) as Hydrogen Peroxide Sensor

Khursheed Ahmad and Haekyoung Kim *

School of Materials Science and Engineering, Yeungnam University, Gyeongsan 38541, Korea; khursheed.energy@gmail.com
*Correspondence: hkkim@ynu.ac.kr

Table of contents

Figure S1. Effectof loading amount of N-rGO on SPCE surface in presence of 0.01 mM H_2O_2 in 0.1 M PBS of pH 7.0 at scan rate of 50 mVs^{-1}.

Figure S2. CV curves of the N-rGO/SPCE in presence of 0.01 mM H_2O_2 in 0.1 M PBS of different pH (3,5,7,9, and 11) at scan rate of 50 mVs^{-1}.

Figure S3. CV curves (**a**) of SPCE in presence of different concentration of H_2O_2 (0.01, 0.05, 0.1, 0.2, 0.3, 0.4, 0.5, 0.6, 0.8, and 1.0 mM) in 0.1 M PBS of pH 7.0 at scan rate of 50 mVs^{-1} and corresponding linear calibration plot (**b**) between peak current response versus concentration of H_2O_2.

Figure S4. CV curves (**a**) of SPCE in presence 0.01 mM H_2O_2 in 0.1 M PBS of pH 7.0 at different applied scan rates (50–500 mVs^{-1}) and corresponding linear calibration plot (**b**) between peak current response versus square root of scan rate.

Figure S5. LSV curves of SPCE in absence (black) and presence (red) of interfering molecules (glucose, chlorophenol, hydrazine, uric acid, urea, dopamine, ascorbic acid, and nitro-phenol) in 0.05 mM H_2O_2 in 0.1 M PBS of pH 7.0 at scan rate of 50 mVs^{-1}.

Figure S6. Chronoamperometry curve (**a**) of N-rGO/SPCE in presence of different concentration of H_2O_2. Calibration curve (**b**) between current response versus concentration of H_2O_2.

Figure S7. Selectivity test of N-rGO/SPCE towrads H_2O_2 determination in presence of various interfering molecules.

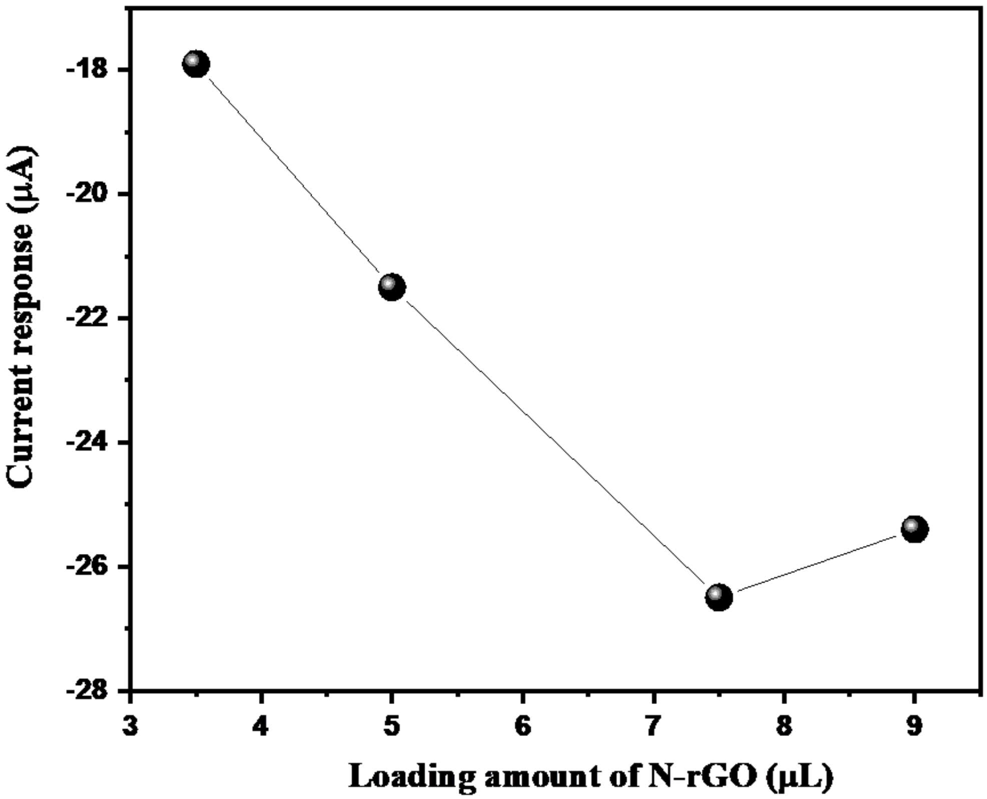

Figure S1. Effectof loading amount of N-rGO on SPCE surface in presence of 0.01 mM H_2O_2 in 0.1 M PBS of pH 7.0 at scan rate of 50 mVs^{-1}.

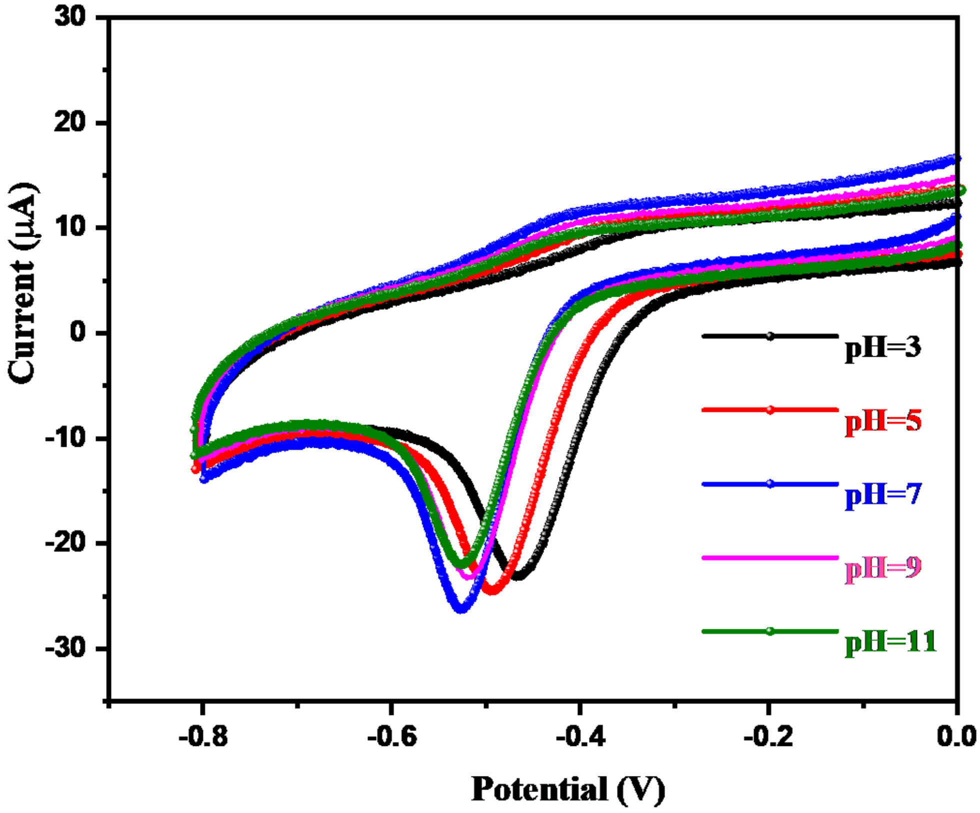

Figure S2. CV curves of the N-rGO/SPCE in presence of 0.01 mM H_2O_2 in 0.1 M PBS of different pH (3,5,7,9, and 11) at scan rate of 50 mVs^{-1}.

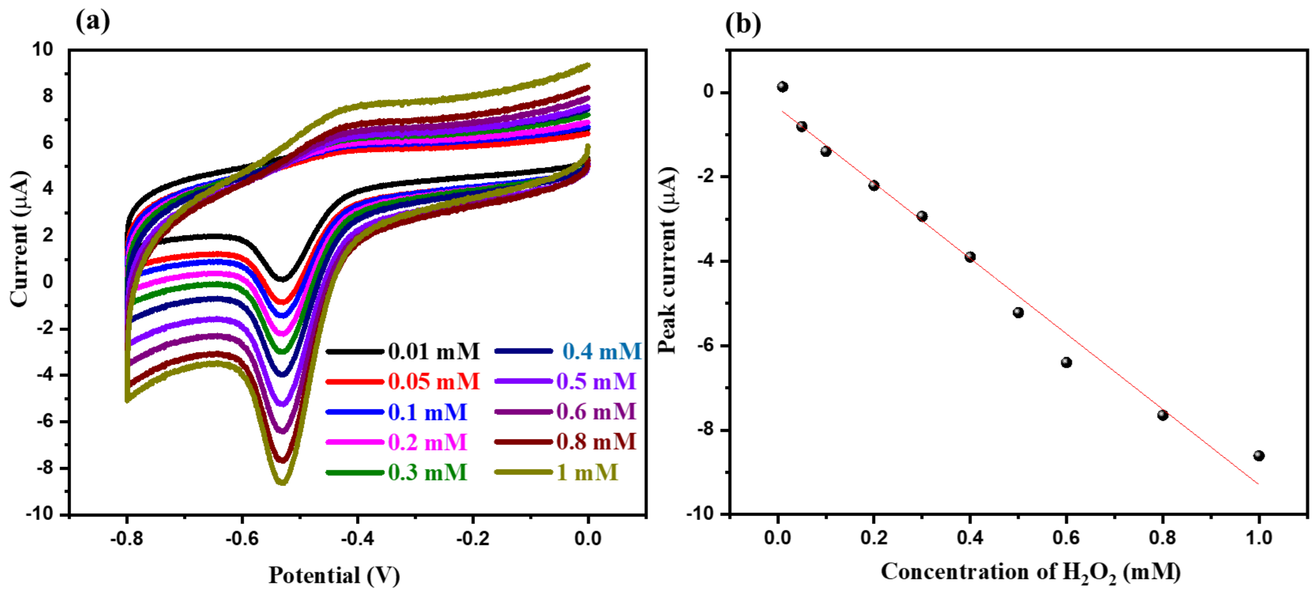

Figure S3. CV curves (**a**) of SPCE in presence of different concentration of H_2O_2 (0.01, 0.05, 0.1, 0.2, 0.3, 0.4, 0.5, 0.6, 0.8, and 1.0 mM) in 0.1 M PBS of pH 7.0 at scan rate of 50 mVs^{-1} and corresponding linear calibration plot (**b**) between peak current response versus concentration of H_2O_2.

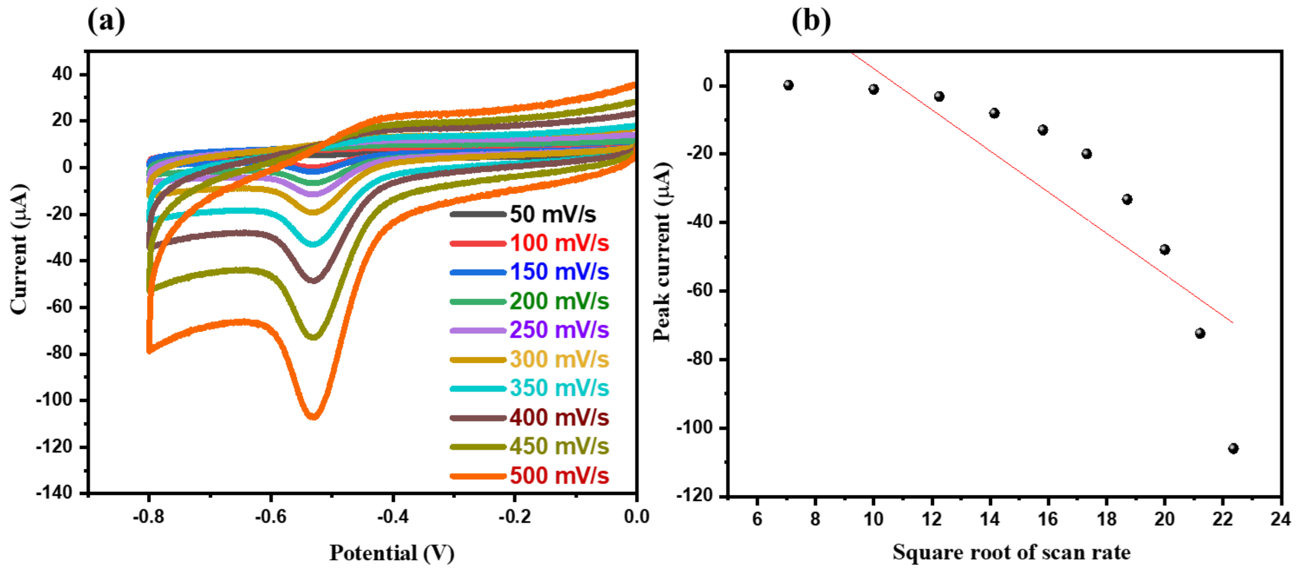

Figure S4. CV curves (**a**) of SPCE in presence 0.01 mM H_2O_2 in 0.1 M PBS of pH 7.0 at different applied scan rates (50-500 mVs^{-1}) and corresponding linear calibration plot (**b**) between peak current response versus square root of scan rate.

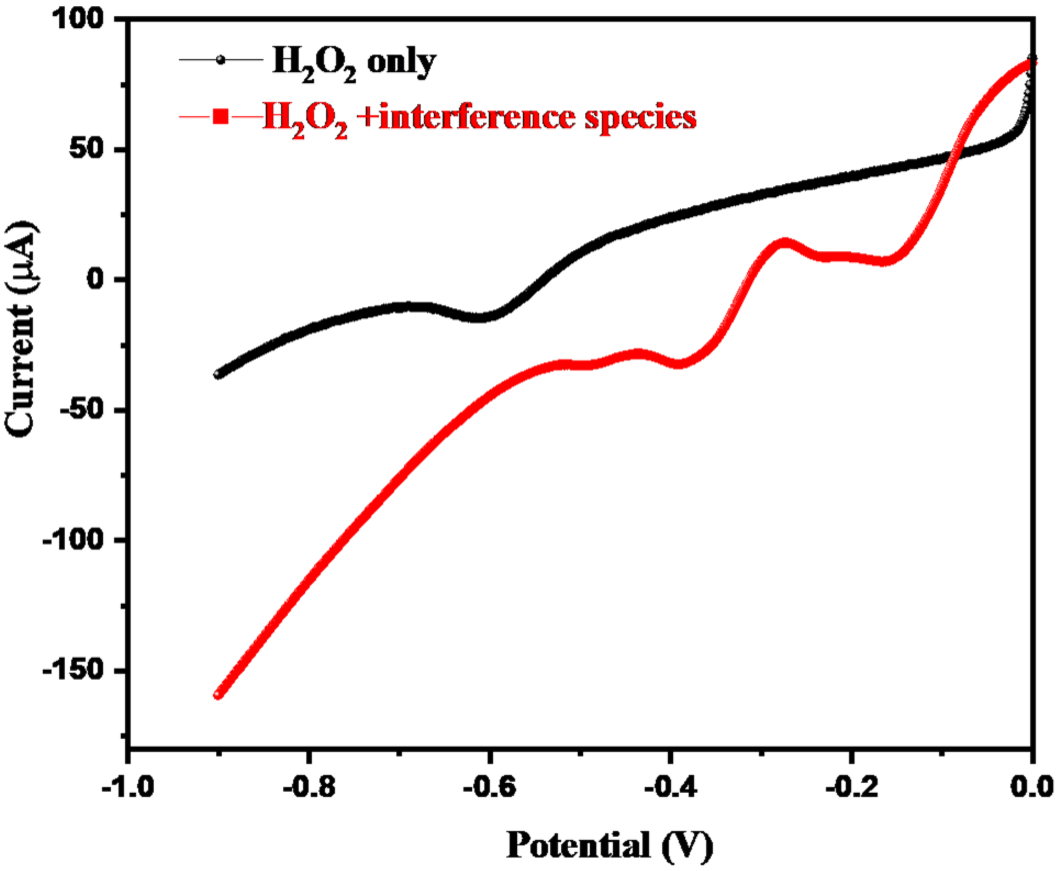

Figure S5. LSV curves of SPCE in absence (black) and presence (red) of interfering molecules (glucose, chlorophenol, hydrazine, uric acid, urea, dopamine, ascorbic acid, and nitro-phenol) in 0.05 mM H_2O_2 in 0.1 M PBS of pH 7.0 at scan rate of 50 mVs^{-1}.

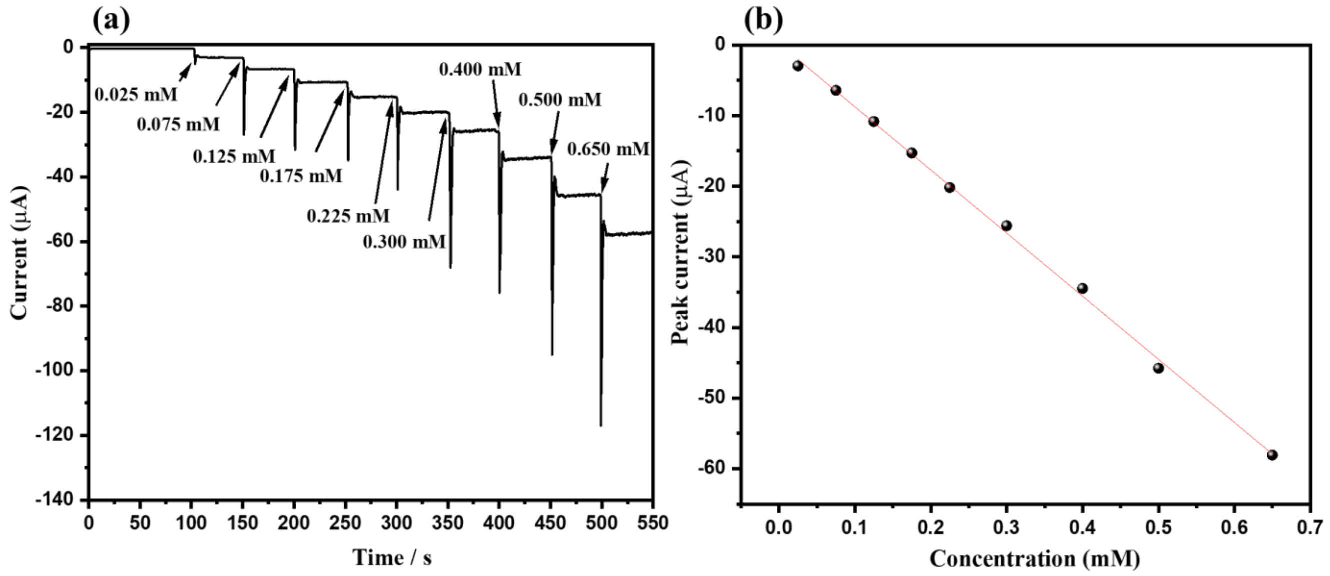

Figure S6. Chronoamperometry curve (**a**) of N-rGO/SPCE in presence of different concentration of H_2O_2. Calibration curve (**b**) between current response versus concentration of H_2O_2.

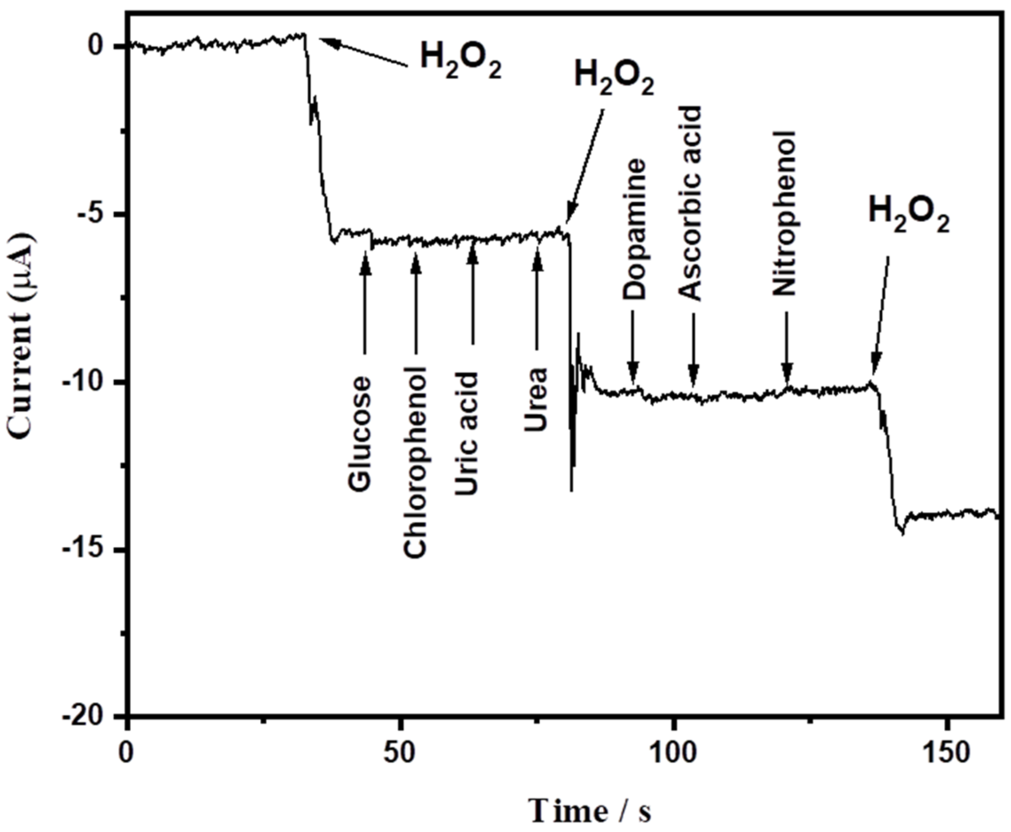

Figure S7. Selectivity test of N-rGO/SPCE towards H_2O_2 determination in presence of various interfering molecules. (concentration of H_2O_2 = 0.05 mM, concnetration of interfereing molecules = 0.25 mM).
